# Porous diaphragm syndrome with recurrent thymoma

**DOI:** 10.1002/rcr2.391

**Published:** 2018-11-28

**Authors:** Takayo Ota, Yoshikazu Hasegawa, Takafumi Okabe, Akira Okimura, Masahiro Fukuoka

**Affiliations:** ^1^ Department of Medical Oncology Izumi City General Hospital Izumi Japan; ^2^ Department of Medical Oncology Nara Hospital Kindai Faculty of Medicine Ikoma Japan; ^3^ Department of Pathology Izumi City General Hospital Izumi Japan

**Keywords:** Ascites, porous diaphragm syndrome, thoracentesis

## Abstract

Porous diaphragm syndrome describes a defect in the diaphragm in which substances pass from the peritoneal cavity to the pleural space. Defects may be congenital or acquired. Acquired defects are caused by the thinning and eventual splitting of collagen fibres in the tendinous part of the diaphragm. We report a case of porous diaphragm syndrome with recurrent thymoma that presented with massive ascites. Increasing intra‐abdominal pressure by ascites and diaphragmatic thinning due to malnutrition by malignancies resulted in the formation of an artificial hole. Thoracentesis changed the balance of hydrostatic pressure, which initiated the influx of a large volume of ascites to the pleural cavity through a hole in the diaphragm.

## Introduction

Porous diaphragm syndrome describes a defect in the diaphragm in which substances, such as fluids, blood, gases, tissue, or exudates, pass from the peritoneal cavity to the pleural space 1. One of the mechanisms of developing a hole in the diaphragm is through increasing intra‐abdominal pressure by ascites. Diaphragmatic thinning may also occur because of malnutrition caused by malignancies, where collagen bundles of the tendinous portion of the diaphragm are broken off 2. In this report, we present a case of porous diaphragm syndrome in which the patient had massive ascites due to recurrent thymoma. Change in hydrostatic pressure gradient across the diaphragm by thoracentesis triggered a sudden onset of dyspnoea caused by the influx of a large volume of ascites into the pleural cavity through a hole in the diaphragm.

## Case Report

**Figure 1:**
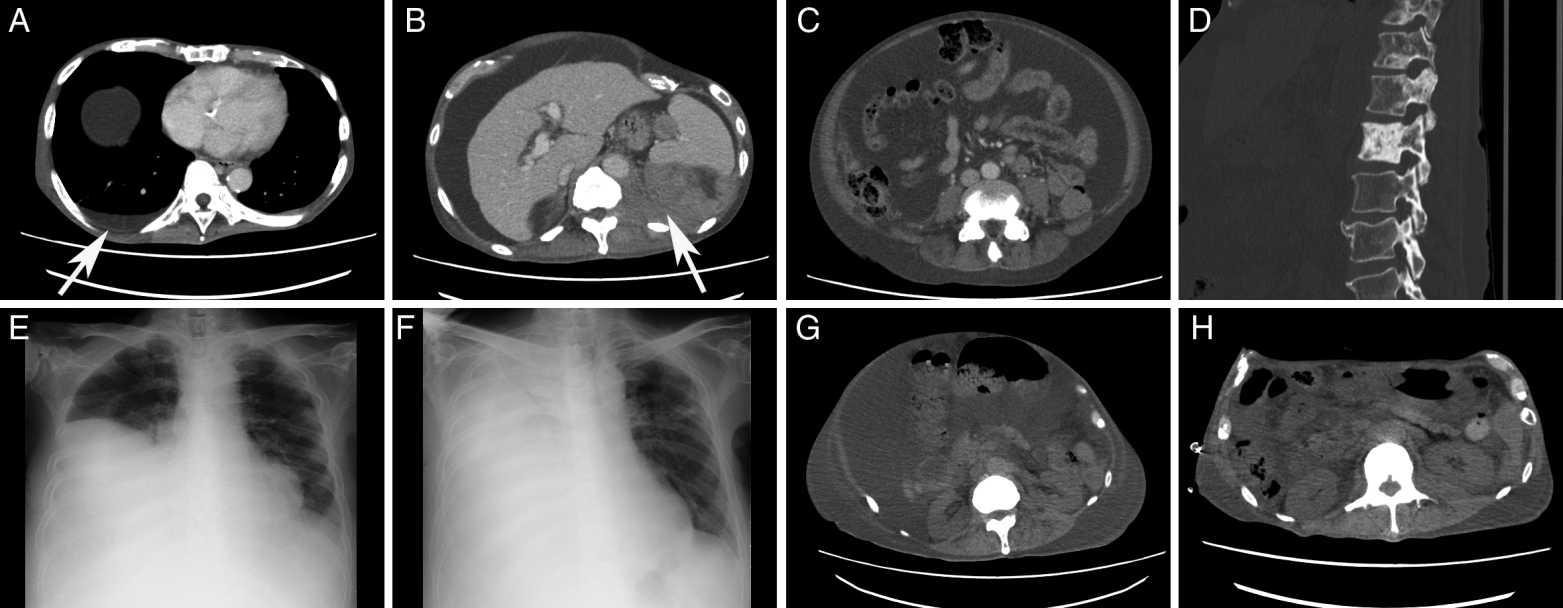
(A) Abdominal computed tomography (CT) scan shows pleural effusion (white arrow); (B) abdominal CT scan shows recurrent thymoma (white arrow); (C) abdominal CT scan shows massive ascites; (D) CT scan shows multiple bone metastases; (E) chest X‐rays show before thoracentesis and (F) 2 h after thoracentesis; and (G) abdominal CT scans show before thoracentesis and (H) after thoracotomy tube insertion. (A, B, C, G, H) Images obtained with mediastinal window settings. (D) An image obtained with bone settings.

A 67‐year‐old male was referred to our hospital in the summer of 2016 in order to control his ascites and leg oedema. He was diagnosed with thymoma in 1992, for which he had an extended thymectomy and received radiotherapy. Until our referral, he had two recurrent episodes. The first one was in 1996. He had an operation for a relapsed tumour and received chemotherapy. The second one was in 2004. He had an extensive operation for a widespread metastasis, but the operation was not able to remove the tumour completely. After the second recurrent episode, he received neither chemotherapy nor radiotherapy. Computed tomography (CT) scan at our referral showed left peritoneal mass, peritoneal dissemination with massive ascites, and multiple bone metastases (Fig. 1). At this time, a small amount of pleural effusion was present (Fig. 1). He was diagnosed with a recurrent type B1 thymoma from a biopsy of the left peritoneal mass.

In September 2016, as a first‐line therapy, the patient was administered carboplatin (AUC 6) on day 1 and paclitaxel (200 mg/m^2^) on day 1 for a 3‐week cycle. On day 6, due to hypo‐gammaglobulinemia, he developed severe pneumonia with febrile neutropenia. He recovered following the administration of antibiotics. In October 2016, a second‐line therapy of everolimus was prescribed at a dose of 5 mg per day, with a reduction to every other day after 15 days due to the development of thrombocytopenia. As right pleural effusion and ascites continued to increase, everolimus was stopped after 1 month.

**Figure 2:**
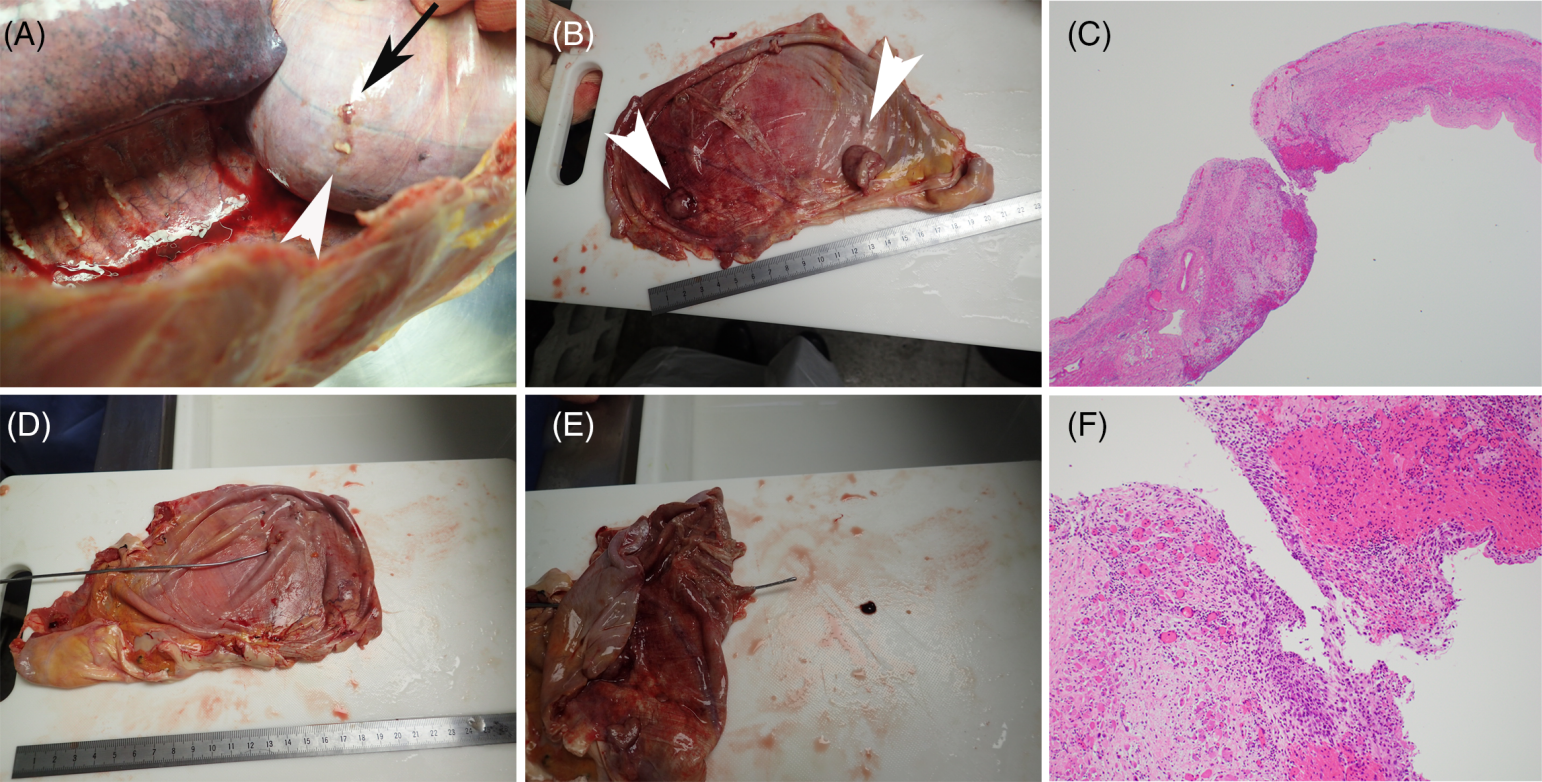
Perforated diaphragm. (A, B, D, E) hole in the diaphragm (black arrow) and metastases (white arrowheads). (C, F) Histopathology of the hole with haematoxylin and eosin staining. No metastasis at the hole. (C) 20×, (F) 100×.

In December 2016, paclitaxel (80 mg/m^2^) was administered every 3–4 weeks as a third‐line therapy. During treatment, the patient’s abdomen gradually became enlarged, and right pleural effusion was increased. After four cycles, thoracentesis was performed, resulting in the removal of 800 mL of fluid. Seventy minutes into of the thoracentesis, the patient presented with acute dyspnoea. He required 7 L of oxygen using a non‐rebreathing mask with a reservoir bag to maintain >90% of SpO_2_. Chest X‐ray showed nearly opacified right hemithorax, and CT scan demonstrated massive pleural effusion (Fig. 1). Immediate tube thoracostomy yielded 10 L of sanguineous fluid. As fluid was removed, the distended abdomen became flat, and dyspnoea was improved. From the next day onwards, fluid in the chest tube decreased but did not stop. OK‐432 (picibanil, Chugai Pharmaceutical Co., Tokyo, Japan) is an immuno‐stimulant obtained from *Streptococcus pyogenes*, which is widely used in Japan for chemical pleurodesis. While pleurodesis with OK‐432 was attempted, it was not effective in stopping fluid from the chest tube. His general condition gradually deteriorated, and he passed away 12 days later. Autopsy examination showed one hole (1 mm diameter) on the right diaphragm, which did not match the position by thoracentesis (Fig. 2). In addition, there were no metastases at the hole, while there were several metastases on the diaphragm (Fig. 2).

## Discussion

Porous diaphragm syndrome is a general term for a group of conditions characterized by an anatomic defect in the diaphragm, through which substances pass from the peritoneal cavity to the pleural space 1. The syndromes can be caused by several different diseases, and in some of the diseases, no obvious defects are observed 1, 2, 3.

The normal diaphragm consists of a central tendinous part and a peripheral muscular part. Diaphragmatic defects mainly occur at the tendinous portion of the central diaphragm and are common in the right hemidiaphragm 1. Even in healthy states, defects may occur more than expected, while in disease states, defects are more apparent 4. Patients may present with single or multiple defects; defects can vary in size from a pinhole up to a centimetre or more in diameter. The defects may be either congenital or acquired.

In addition to porosity at the right hemidiaphragm, right‐side predominance of this syndrome is explained by the following three reasons 1, 2. First, peritoneal circulation produced by intestinal peristalsis is the preferential flow of peritoneal fluids from the pelvis to the right upper quadrant. Second, pressure is highest at the pelvis and lowest at the right upper quadrant during inspiration. Third, the contracting diaphragm laid atop the relatively firm liver acts as a piston and generates unidirectional flow from the abdominal to the pleural cavity.

In the present case, it is difficult to determine when the defect first appeared in the diaphragm. Pleural effusion was present when the recurrent thymoma was diagnosed. Right pleural effusion accumulated during the second‐line treatment. Removing pleural effusion by thoracentesis triggered the formation of a defined hole. Autopsy demonstrated that the underlying condition for the syndrome was not metastasis of the tumour but massive ascites. The hole might have originated from a small defect when right pleural effusion was initiated.

The ultimate goal of the treatment is to seal defects. Pleurodesis or video‐assisted thoracic surgery might be an option to achieve this 5. A chest tube is used for the relief of dyspnoea, but it induces loss of massive fluids, and once it is placed, it is difficult to remove.

In summary, we describe a case of porous diaphragm syndrome with thymoma. This case indicates that any instance of a high‐pressure gradient between abdominal cavity and pleural space combined with a pre‐existing diaphragmatic weakness or defect may predispose to this condition.

### Disclosure Statement

Appropriate written informed consent was obtained for publication of this case report and accompanying images.
